# Integrative Phenotyping of Knee Osteoarthritis: Linking WOMAC Cut-Offs, Kellgren–Lawrence Grades, and Cluster Analysis for Personalized Care

**DOI:** 10.3390/life15101542

**Published:** 2025-10-01

**Authors:** Ciprian-Vasile Pojala, Marius Alexandru Moga, Cristiana-Elena Pojala, Nadinne Alexandra Roman, Radu Dan Necula, Sebastian Ionut Toma, Rosana Mihaela Manea, Lorena Dima

**Affiliations:** 1 Faculty of Medicine, Transilvania University of Brasov, 500001 Brasov, Romania; ciprian.pojala@unitbv.ro (C.-V.P.); rosana.manea@unitbv.ro (R.M.M.);; 2 Department of Radiology and Medical Imaging, University of Medicine and Pharmacy of Craiova, 200349 Craiova, Romania

**Keywords:** clustering, WOMAC, osteoarthritis, cut-off, phenotypes, personalized management

## Abstract

Knee osteoarthritis (OA) is a complex condition with varying pain, functional limitations, and structural changes. Traditional classification using radiographic grades may not fully reflect individual patient experiences. This study aimed to establish WOMAC score cut-offs for KL grades and identify knee OA phenotypes through cluster analysis in a cohort of 99 adults, examining functional and radiological status, factors such as age, sex, body mass index (BMI), comorbidities, and psychological status. Receiver operating characteristic (ROC) analysis helped establish WOMAC cut-off scores related to KL grades, and cluster analysis identified phenotypic subgroups. The analysis showed that higher WOMAC scores correlated with advanced KL grades, leading to a five-tier classification of symptomatic severity: minimal or no symptoms (≤24), mild (25–41), moderate (42–69), severe (70–86), and extreme (≥87). Cluster analysis identified four distinct phenotypic groups: (1) younger patients exhibiting minimal symptoms and low KL grades; (2) individuals with moderate disease are characterized by functional deficits; (3) patients presenting with moderate-to-severe symptoms and significant joint narrowing; and (4) a subgroup experiencing severe pain, high levels of disability, advanced KL grades, elevated psychological distress, and an increased BMI. The study supports WOMAC cut-offs as key indicators of knee OA severity and shows that cluster analysis can reveal distinct phenotypes, underscoring the need for personalized management strategies in knee OA treatment.

## 1. Introduction

Knee osteoarthritis (OA) is a progressive joint disorder that leads to significant mobility impairment, affects functional independence, and diminishes overall quality of life [1]. The heterogeneous nature of OA presents a challenge, as there is frequently a divergence between the symptoms experienced by patients and the radiographic severity of the condition. This variability complicates both diagnosis and treatment planning, as individual patients may exhibit distinct underlying mechanisms of disease and diverse clinical presentations [2]. Current management of knee OA is largely symptom-focused, emphasizing lifestyle modifications (e.g., exercise, weight loss) and analgesic or anti-inflammatory medications, as no cure or disease-modifying therapy exists [3]. Considering the misalignment between clinical and radiological evaluations, it is essential to implement personalized treatment strategies grounded in a precise classification of disease severity to enhance patient safety and optimize therapeutic outcomes [4].

Recent studies have shown that radiographic severity assessed by Kellgren–Lawrence (KL) grade does not always correlate with the intensity of pain, functional limitations, or disability reported by patients [5,6,7]. Some individuals with severe radiographic changes report minimal symptoms, while others with mild radiological findings may experience severe pain and functional impairment. This discordance has been well documented in both cross-sectional and cohort studies, emphasizing the need for multidimensional patient assessment [8].

Traditional “one-size-fits-all” approaches and clinical trials that group all OA patients, typically defined by knee pain and radiographic changes, fail to acknowledge individual variability [9,10]. Historically, OA management protocols have mainly overlooked the significant heterogeneity in patient symptoms, comorbidities, and disease progression, thereby limiting the effectiveness of such universal interventions. Recent research has highlighted the need for stratified or phenotype-based care, reflecting a shift away from uniform treatment protocols toward more targeted, personalized management strategies [11,12]. Research suggests at least six clinical phenotypes, including inflammation-dominant, metabolic syndrome-related, biomechanical overload, chronic pain, bone and cartilage metabolism, and minimal joint disease depressive phenotype. Defining these phenotypes is expected to clarify differences in pathophysiology and prognosis between subgroups, enabling clinicians to tailor interventions to specific profiles and enhance the likelihood of treatment success [10,13]. Five structural phenotypes have been proposed: the inflammatory, meniscus cartilage, subchondral bone, and atrophic and hypertrophic phenotypes [14].

Improving care for knee OA requires integrating radiographic, clinical, and functional assessments to understand the disease in individual patients better. Diagnosis and staging have traditionally relied on X-ray evaluations, such as the Kellgren–Lawrence (KL) grading system, to identify structural damage. However, structural severity on imaging does not always correlate with symptom severity or functional impairment. Many patients may experience significant pain and limited mobility despite mild X-ray changes, while others with more severe radiographic evidence may report minimal pain [15]. Previous research has identified a significant relationship between the radiological grade of knee osteoarthritis, as assessed by the KL grade, and the functional evaluation level of patients with osteoarthritis, as measured by the Western Ontario and McMaster Osteoarthritis (WOMAC) questionnaire. The correlation between those two variables is not fully explained [16].

Additionally, WOMAC is a widely used patient-reported outcome measure that assesses pain, stiffness, and functional limitations in knee osteoarthritis (OA). However, its clinical relevance as a threshold-based tool for guiding treatment decisions remains underexplored [17]. Furthermore, in clinical practice, using WOMAC, merely obtaining a WOMAC score is not enough—it is essential to interpret that score against meaningful benchmarks. Establishing cut-off values for the WOMAC index can significantly guide treatment strategies. Recent research revealed a WOMAC score of approximately 82.8, which was identified as the threshold above which patients considered their symptom state acceptable following knee arthroplasty [18]. Similarly, the KL grading system, a radiographic classification of OA, does not fully capture the functional impairments experienced by patients. Thus, combining symptom-based and imaging-based assessments could enhance patient stratification and optimize safety-oriented therapeutic intervention [17].

While the KL grade is considered the standard for radiographic assessment of OA and is an invaluable tool for surgeons, particularly when considering invasive treatment options, the cut-off for the radiographic diagnosis of OA by grade KL may exclude some individuals [19,20]. Research on Caucasian populations to assess the level of dichotomy or synchrony between KL grading and the expression of clinical symptoms produced more results [21].

Emerging evidence indicates that distinct OA phenotypes differ not only in underlying pathophysiological mechanisms but also in trajectories of disease progression, treatment responsiveness, and long-term patient-reported outcomes. For instance, inflammation-dominant phenotypes may benefit more from targeted anti-inflammatory strategies, whereas metabolic phenotypes often require integrated weight management and cardiovascular risk control alongside joint-directed therapy [10,11,12,14]. Stratifying patients into clinically meaningful phenotypes has been associated with improved prediction of disability progression and more efficient treatment selection, ultimately enhancing functional outcomes and quality of life [11,14]. These advances support the transition from “one-size-fits-all” approaches toward precision rehabilitation and phenotype-informed care pathways in knee osteoarthritis.

Building on recent advancements, our study presents an integrative approach that combines radiographic severity, measured using KL grades, with patient-reported outcomes from the Western Ontario and McMaster Universities Osteoarthritis Index (WOMAC) and various psychological and metabolic comorbidities, such as anxiety, depression, body mass index (BMI), and hypertension. This methodology not only calibrates WOMAC scores in relation to structural damage but also identifies specific patient subgroups whose disease burden may be worsened by psychosocial or metabolic factors. To our knowledge, this is the first phenotyping study to explicitly link KL-associated WOMAC cut-offs with clustering based on psychological variables. This approach has the potential to enhance patient stratification, surpass current phenotype classifications, and inform more personalized treatment strategies.

The primary aim of this study is to identify distinct patient subgroups and optimize therapeutic strategies for patient safety, based on the clinical, functional, and radiological characteristics of patients with knee osteoarthritis.

The secondary aims address the potential use of the WOMAC scale as an indicator of knee OA severity and to investigate psychological and metabolic factors influencing OA progression, including anxiety, depression, body mass index, hypertension, and diabetes mellitus, to determine their association with disease severity.

## 2. Materials and Methods

### 2.1. Study Design: Participants

This observational cross-sectional study enrolled 99 participants diagnosed with knee OA based on clinical assessment and radiographic evidence, classified according to the KL grading system. Patients were consecutively recruited from the Medlife Brașov outpatient clinic between March 2023 and February 2024. The age range of included participants was set at 18–90 years to capture both typical degenerative cases and rare early-onset presentations, such as those related to genetic predispositions or previous joint trauma. Age was subsequently analyzed as a continuous variable and incorporated into the clustering analysis to reduce bias arising from heterogeneous age groups. Although some studies have restricted recruitment to participants aged ≥45 years, our broader range aimed to improve external validity by reflecting the real-world spectrum of knee OA cases.

As a robustness check, all primary analyses were re-examined after restricting the cohort to participants aged 45 years or older; the pattern of results (ROC AUCs, optimal cut-offs, and cluster structure) remained unchanged within the corresponding 95% CIs. No participants met the criteria for under-nutrition (BMI < 18.5 kg/m^2^) at enrollment. We lacked formal socio-economic metrics; this is acknowledged as a limitation, and future studies will incorporate standardized SES and occupational exposure assessments.

Exclusion criteria were: neurological disorders (stroke, Parkinson’s disease, multiple sclerosis); secondary OA (congenital malformations, avascular necrosis, post-infectious arthritis); autoimmune inflammatory diseases (rheumatoid arthritis, psoriatic arthritis, systemic lupus erythematosus, or arthritis associated with inflammatory bowel disease); chronic opioid therapy (potentially influencing pain scores); and inability to undergo radiographic assessment.

Nutritional or socio-economic status was not used as an exclusion criterion, as no participants demonstrated undernutrition (BMI < 18.5 kg/m^2^), and severe socio-economic deprivation was not observed in this urban outpatient setting. Nonetheless, the absence of systematic evaluation of nutrition and socio-economic status is acknowledged as a limitation. Occupation and daily workload were also not used as criteria, but may influence disease onset and progression; this should be addressed in future studies.

The study protocol was approved by the Ethics Committee of Transilvania University of Brașov (Approval No. 3, dated 10 February 2023) and complied with the principles outlined in the Declaration of Helsinki.

### 2.2. Clinical Outcome Measures

Clinical status was assessed using validated instruments administered by a trained physician.

Psychological status was assessed using the Hospital Anxiety and Depression Scale (HADS), as a self-rating tool. It has 14 validated items comprising two subscales for anxiety and depression (7 items each). Each item is scored from 0 to 3, resulting in subscale scores ranging from 0 to 21. Scores of 0–7 are considered normal, 8–10 indicate borderline abnormal cases, and ≥11 suggest clinically significant anxiety or depression [22].

Western Ontario and McMaster Universities Osteoarthritis Index (WOMAC): a 24-item questionnaire with three subscales: Pain (5 items, score 0–20), Stiffness (2 items, score 0–8), and Physical Function (17 items, score 0–68). Each item is scored on a Likert scale from 0 (“none”) to 4 (“extreme”), giving a total score of 0–96, with higher scores indicating more severe symptoms [23]. In this study, the WOMAC total score was analyzed on its original 0–96 scale without normalization to 0–100. Thresholds derived from ROC analyses correspond directly to raw WOMAC total scores.

Lequesne Algofunctional Index: a composite measure assessing pain, walking distance, and daily activities, yielding scores from 0 (no disability) to 24 (severe disability) [24].

Visual Analog Scale (VAS): pain intensity was measured using a 0–10 cm line, where 0 represented “no pain” and 10 represented “worst imaginable pain” [25].

### 2.3. Metabolic Assessments

Metabolic comorbidities were assessed at the same visit. Body mass index (BMI) was calculated as weight in kilograms divided by height in meters squared (kg/m^2^), measured using standardized clinic procedures. Hypertension was defined according to international criteria as systolic blood pressure ≥ 140 mmHg and/or diastolic blood pressure ≥ 90 mmHg, or current antihypertensive treatment, and was diagnosed by a previous specialist physician. Diabetes mellitus was diagnosed by a prior physician diagnosis or ongoing use of antidiabetic medication. Data were cross-verified with medical records.

### 2.4. Radiographic Assessments

The KL classification grade of knee osteoarthritis severity ranges from 0 (no OA) to 4 (severe OA) [26].

Radiographic severity was independently graded by two fellowship-trained musculoskeletal radiologists, each with over 10 years of experience, who were blinded to the patient’s clinical information.

The KL classification was applied according to established criteria: grade 0 = no radiographic features; grade 1 = doubtful joint space narrowing and possible osteophytic lipping; grade 2 = definite osteophytes and possible joint space narrowing; grade 3 = multiple osteophytes, definite joint space narrowing, sclerosis, possible bony deformity; grade 4 = large osteophytes, marked joint space narrowing, severe sclerosis, and definite bony deformity [26]. Disagreements between readers were resolved by consensus, and inter-observer agreement was assessed on a random 20% sample, yielding a Cohen’s kappa coefficient of 0.81, indicating excellent reliability. The KL scale remains the international gold standard for both clinical and epidemiological studies and was therefore chosen in the present research [27,28].

Standardized weight-bearing anteroposterior (AP) knee radiographs (fixed-flexion protocol) and lateral views were acquired using digital detectors according to clinic protocol; patellofemoral views were available when clinically indicated. For inter-reader agreement, a random 20% sample was double-graded; Cohen’s κ = 0.81 (95% CI–) indicated excellent reliability. When both knees were available, the index knee was defined a priori as the more symptomatic side (as measured by VAS) or, if equal, the knee with the higher KL grade; only the index knee entered the analyses to preserve independence.

### 2.5. Statistical Analysis

All statistical analyses were conducted in IBM SPSS Statistics version 26.0 (IBM Corp., Armonk, NY, USA). Enhanced visualizations, including ROC curves, annotated boxplots, violin plots, and radar cluster profiles, were created using Python 3.11 (scikit-learn, matplotlib, seaborn). Descriptive statistics are reported as mean ± SD for continuous variables and n (%) for categorical variables.

Receiver operating characteristic (ROC) analyses were performed to evaluate the discriminative performance of the WOMAC total score against KL radiographic grading (the gold standard reference for structural severity). AUCs were computed with 95% confidence intervals using the nonparametric DeLong method. Optimal thresholds were identified using Youden’s J index. Sensitivity and specificity at each cut-off are reported with Wilson score confidence intervals (Supplementary Materials, Figure S1 and Table S1). To assess robustness and correct for optimism due to sample reuse, we applied a bootstrap -validation with 1000 resamples and the 0.632 estimator [29]. Apparent and optimism-corrected AUCs, as well as the stability of Youden thresholds across resamples (median and interquartile range), are presented in Supplementary Table S5.

**Figure 1:**
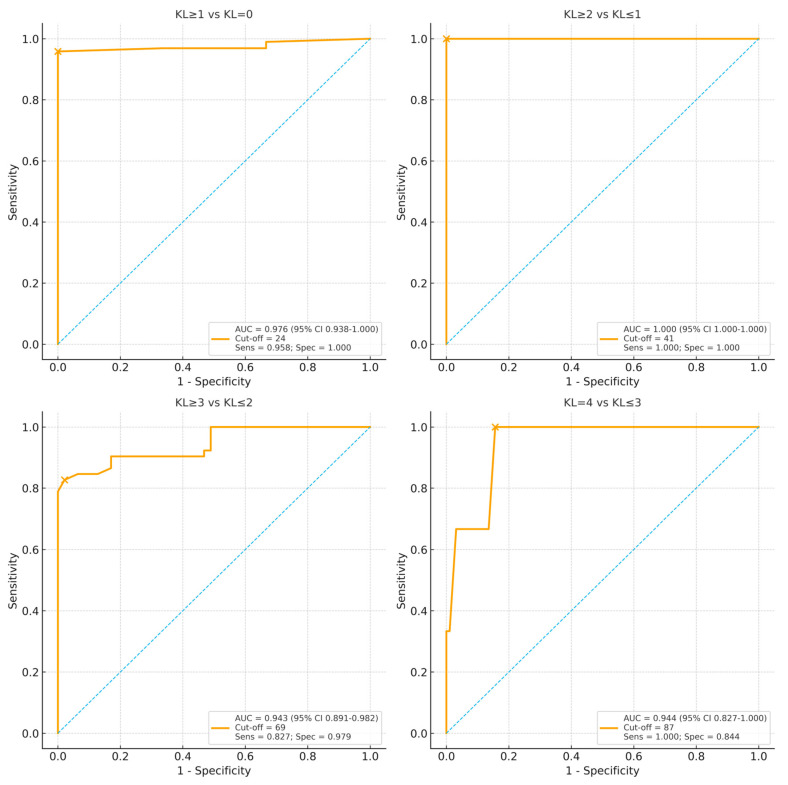
ROC curves for WOMAC Total vs. KL grades. Panels display AUC (95% CI, DeLong). Optimal cut-offs (Youden’s J) are annotated on each panel; full metrics (AUCs and 95% CIs, sensitivity and specificity with 95% CIs) are in Supplementary Table S1.

**Figure 2:**
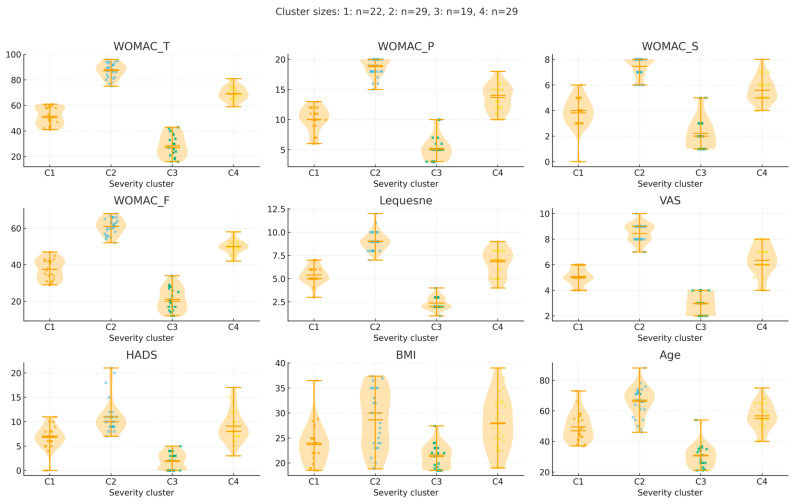
Distribution of clinical and demographic variables across the four severity-based clusters.

Clustering analyses were performed to identify clinically distinct subgroups of patients. Variables included WOMAC total and subscores (Pain, Stiffness, Function), Lequesne Index, VAS, HADS, BMI, age, and radiographic features (KL grade, joint space narrowing, osteophytes). Continuous variables were z-standardized, and ordinal radiographic variables were treated as continuous, a common approach in phenotyping. K-means clustering was performed with k-means++ initialization, Euclidean distance, 50 random starts, and up to 1000 iterations. The Elbow criterion and clinical interpretability determined the number of clusters (k = 4). Internal validity was assessed using silhouette coefficients (average = 0.502), and cluster stability was examined through 200× subsampling with Jaccard similarity indices [30,31,32]. Detailed results with standardized cluster profiles are shown in Figure 1, Figure 2 and Supplementary Table S2.

Between-cluster differences and radiographic characteristics across clusters, one-way ANOVA was applied when the assumptions were met, specifically normality (as determined by the Shapiro–Wilk test) and homoscedasticity (as determined by Levene’s test). When assumptions were violated, Welch’s ANOVA or the Kruskal–Wallis test was used. Post hoc analyses employed Tukey’s HSD for parametric contrasts and Dunn–Bonferroni for nonparametric contrasts. Effect sizes are reported as η^2^ or ω^2^ for ANOVA, ε^2^ for Kruskal–Wallis, and Hedges’ g for pairwise comparisons (Supplementary Table S3). Categorical associations, such as hypertension distribution across clusters, were analyzed with chi-square tests, supplemented by standardized residuals (Supplementary Table S4).

## 3. Results

### Descriptive Analysis

In Table 1, a descriptive analysis of the study population’s characteristics is presented.

Figure 1 presents the ROC analysis performed on Kellgren–Lawrence grading and WOMAC total scores. The analysis of knee OA severity using the ROC curve assessment demonstrated a strong relationship between the KL classification and WOMAC total scores. The area under the curve (AUC) values ranged from 0.943 to 1000, indicating high diagnostic accuracy across KL grades. Sensitivity and specificity varied with increasing disease severity, with the WOMAC total score thresholds identifying significant functional impairment at different KL stages.

ROC curve analyses demonstrated excellent discriminatory ability of the WOMAC total score across Kellgren–Lawrence (KL) thresholds (Figure 1). The area under the curve (AUC) values were 0.976 (95% CI: 0.938–1.000) for KL ≥ 1 vs. 0, 1.000 (95% CI: 1.000–1.000) for KL ≥ 2 vs. ≤1, 0.943 (95% CI: 0.892–0.980) for KL ≥ 3 vs. ≤2, and 0.944 (95% CI: 0.832–1.000) for KL = 4 vs. ≤3. Optimal Youden J cut-offs for the WOMAC total score were 24, 41, 69, and 87, respectively. At these thresholds, sensitivities ranged from 0.827 to 1.000 and specificities from 0.844 to 1.000, indicating both high diagnostic accuracy and consistent separation between adjacent KL categories. Full results, including 95% confidence intervals for sensitivity and specificity, are provided in Supplementary Table S1.

Bootstrap internal validation using the 0.632 estimator with 1000 replications yielded optimism-corrected AUC values that were nearly identical to the apparent estimates, and Youden’s J thresholds remained stable across resamples (Supplementary Table S5).

Following the ROC curve analysis, a differentiation in WOMAC total scores led to a secondary classification of scoring, as shown in Table 2. The following table summarizes the interpretation of WOMAC total scores according to KL radiographic grading. Score intervals are based on the optimal cut-off values identified using Youden’s J statistic from ROC curve analysis, ensuring the best balance between sensitivity and specificity. The results suggest specific cut-off points and intervals of WOMAC scores associated with KL radiological grading, indicating the severity of knee OA.

The hierarchical clustering analysis revealed that the optimum number of clusters is 3 to 5. For the K-means clustering, the Elbow method indicated that the optimum number of clusters is 4. The K-means clustering identified 4 clusters (as possible clinical and radiological phenotypes). The results of the ANOVA analysis showed all considered variables are statistically significant (*p* < 0.001). The descriptive analysis of each cluster is provided in Table 3.

Cluster analysis using standardized WOMAC, Lequesne, VAS, HADS, BMI, and age identified four stable patient subgroups (n = 22, 29, 19, and 29, respectively). Standardized profiles of each cluster are shown in Figure S1 (Supplementary Materials), illustrating distinct severity patterns. Internal validity was confirmed by a silhouette coefficient of 0.502 and by subsampling stability analysis (median Jaccard similarity per cluster 0.61–0.74; Supplementary Table S2).

In cluster 1, the subjects might experience slight discomfort and occasional stiffness, causing minimal disruptions to their daily activities. In cluster 2, Individuals face a growing challenge of pain and functional limitations, with the emergence of noticeable joint wear and tear. Clusters 3 and 4 suggest persistent pain, significant functional limitations, and severe joint deterioration.

Further analysis using one-way ANOVA was conducted to determine whether statistically significant differences existed among the knee OA clusters identified through K-means clustering based on clinical parameters.

The ANOVA results revealed significant differences among the clusters for multiple clinical variables, including WOMAC Total Score (F(3, 95) = 242.36, *p* < 0.001), WOMAC Pain Score (F(3, 95) = 208.10, *p* < 0.001), WOMAC Stiffness Score (F(3, 95) = 100.18, *p* < 0.001), and WOMAC Functional Score (F(3, 95) = 193.34, *p* < 0.001). Additionally, joint space narrowing (F(3, 95) = 102.99, *p* < 0.001) revealed highly significant differences across clusters. These findings suggest that disease severity increases consistently across clusters.

Between-cluster differences were evaluated after verifying assumptions (Shapiro–Wilk, Levene). Where needed, Welch or Kruskal–Wallis tests corroborated inferences (see Supplementary Table S4). Results remained consistent across approaches. We also report effect sizes (η^2^/ω^2^ or ε^2^; pairwise Hedges’ g) to quantify the magnitude of differences (Supplementary Materials Table S4).

A post hoc Tukey HSD test was conducted to investigate pairwise differences between clusters further. The results indicated that:

- WOMAC Total Score: Mild OA patients had significantly lower scores than all other groups (*p* < 0.001), confirming increasing severity.
- WOMAC Pain Score: Mild OA cluster differed significantly from Moderate, Moderate-Severe, and Severe OA (*p* < 0.001).
- WOMAC Functional and Stiffness Scores: Stepwise increases were observed across groups, all of which were statistically significant (*p* < 0.001).
- Joint Space Narrowing and Osteophyte Scores: Progressively higher in more severe clusters (*p* < 0.001).
- BMI: Clusters 3 and 4 had significantly higher BMI than Clusters 1 and 2 (*p* < 0.001), consistent with a metabolic phenotype in more severe OA.

To facilitate detailed comparisons, a comprehensive table of post hoc pairwise comparisons for all clinical variables is provided as Supplementary Materials Table S6.

A chi-square test for independence was conducted to examine the relationship between hypertension and knee OA clusters. The association between hypertension and cluster severity was statistically significant (χ^2^(3) = 31.28, *p* < 0.001). Examination of standardized residuals indicated that patients in Severity cluster 1 had a markedly lower prevalence of hypertension than expected, while those in cluster 4 showed a higher prevalence, confirming a strong relationship between cardiovascular comorbidity and OA severity profiles.

Violin plots illustrate the distribution of WOMAC total and subscales (Pain, Stiffness, Function), Lequesne Index, VAS, HADS, BMI, and age across the four K-means–derived severity clusters (C1–C4). Individual data points are overlaid (jittered), and horizontal lines represent cluster medians. Cluster sizes: C1 = 19, C2 = 22, C3 = 29, C4 = 29, as shown in Figure 2.

Diabetes Mellitus was not statistically significant in our analyses; therefore, no results are presented.

## 4. Discussion

### 4.1. Clinical Interpretation of WOMAC Cut-Offs and KL Grades

This study highlights the importance of multidimensional assessment in knee osteoarthritis (OA), reinforcing the need for patient safety and tailored therapeutic interventions. Our findings confirm a strong correlation between WOMAC cut-off values and KL grades, allowing an evidence-based classification of OA severity. The ROC curve analysis demonstrates the high diagnostic accuracy of this approach, providing a practical tool for clinical decision-making.

These results should be interpreted in the context of an observational study, with a cross-sectional design and moderate sample size. Although the WOMAC thresholds and subgroups identified by clustering suggest clinical utility, the cross-sectional nature limits causal inferences. Their value is primarily hypothesis-generative and may guide the design of interventional studies (e.g., targeted selection of patients by phenotype/severity and definition of inclusion thresholds), as well as the development of personalized clinical pathways in rehabilitation and pain management.

Our results confirm a progressive increase in KL grade from Cluster 1 (Mild OA) to Cluster 4 (Severe OA). As expected, WOMAC total scores, pain levels (VAS), and functional impairment worsen with increasing OA severity. However, the presence of metabolic and psychological comorbidities (BMI, hypertension, anxiety, and depression) in more severe clusters suggests that knee OA progression is influenced by more than just structural joint damage. To our knowledge, this is the first study to propose clinically relevant WOMAC cut-off values directly correlated with KL grades and to validate OA phenotypes through clustering that includes psychological comorbidities (e.g., anxiety/depression). These findings support previous studies showing a significant association between WOMAC scores and KL grades, particularly for KL grades of 3 or higher, where structural severity is associated with greater functional impairment [33,34].

### 4.2. Psychological and Metabolic Influences on OA Severity

Pain in OA is not purely mechanical; it is significantly influenced by psychological status and metabolic factors. Pain perception varies and does not always align with radiographic severity, highlighting the need for symptom-based classification [21,35,36,37]. Cultural and individual factors also influence pain tolerance, which can fluctuate and impact daily function in an inconsistent manner [38]. Beyond radiographic findings, our clustering analysis identified that more advanced OA phenotypes were frequently associated with higher body mass index (BMI), hypertension, and psychological comorbidities, such as anxiety and depression.

Pain perception in OA is influenced by psychological factors, particularly depression, which can heighten sensitivity and lead to disproportionate pain even in early disease stages [39]. Studies have also linked KL grading with depression in knee OA patients [40]. In our analysis, Cluster 4 reflects a severe OA depressive phenotype, where obesity and psychological distress likely amplify pain and disability. These patients may benefit from integrated interventions, including weight loss and cognitive behavioral therapy. Obesity increases joint load and accelerates cartilage damage, contributing to higher pain severity in OA [41]. Our clustering results suggest that obesity and pain are associated; therefore, metabolic and cardiovascular factors should be addressed in treatment, therapy, and pharmacological management plans.

Hypertension may exacerbate OA symptoms by impairing vascular supply and joint repair, potentially affecting WOMAC scores. Reduced blood flow can lead to a nutrient deficiency in joint tissues, contributing to pain and functional decline [42]. Effective data processing is essential for optimizing time and achieving consensus on medical criteria. There is considerable disagreement concerning the KL score for early OA classification. Luyten et al. (2018) highlighted the variability of grade 2 scoring across centers and recommended using a KL grade of 0 or 1 in the classification criteria due to the absence of better alternatives [43]. Mahmoudian et al. restricted these classification criteria to subjects with KL grade 1, based on prior reports indicating a strong association between KL grade 1 and an increased risk of developing radiographic knee osteoarthritis (OA) [44]. Migliore et al. established that any radiographic changes in symptomatic patients should be regarded as established disease rather than early disease, thus excluding KL > 0 from their criteria [45]. Our research findings suggest that the WOMAC score and the KL grading system could benefit from further stratification. This would facilitate more effective and targeted treatment for patients with knee osteoarthritis (OA) while also accounting for the varying stages of progression from one severity level of the disease to another.

### 4.3. Inflammation, Depression, and OA Phenotype Overlap

Our cluster results differ not only in pain and function but also in associated comorbidities: more advanced OA clusters tend to have higher BMI, hypertension, and greater prevalence of anxiety and depression. Recent studies suggest that chronic low-grade inflammation may be a shared mechanism underlying the connection between joint degeneration and cognitive and affective disturbances in OA. Our findings support this hypothesis, given the overlap between severe OA clusters and high HADS scores. Studies exploring interconnected pathways between inflammation, pain, and cognitive decline in OA support this connection, highlighting inflammation as a shared driver of both physical and psychological deterioration [46]. Additionally, evidence from autoimmune conditions such as SLE reveals that inflammatory and thrombotic pathways significantly contribute to neuropsychiatric manifestations, including depression and anxiety, reinforcing the broader relevance of inflammation-driven processes across chronic rheumatic diseases [47]. This opens the door for future OA phenotyping studies that include inflammatory biomarkers, such as CRP or IL-6, to refine clusters and guide targeted therapy. The results from our research highlight the need for a personalized, multidimensional management approach rather than a one-size-fits-all treatment.

To avoid over-simplifying the conclusions, we present these findings as exploratory, not definitive, recognizing that a cross-sectional study cannot demonstrate causality. References to mechanisms (e.g., inflammatory components and structure–symptom discordance) are aligned with the review literature and serve to generate hypotheses for future testing [46,47,48,49].

### 4.4. Personalized Management Strategies for Identified Clusters

#### 4.4.1. Non-Pharmacological Interventions

Non-pharmacological interventions form the cornerstone of OA management, with exercise and physiotherapy recognized as first-line therapies that significantly improve pain, physical function, and stiffness [50,51]. Tailored exercise programs focusing on muscle strength, joint mobility, and adherence are recommended [52]. Weight management, particularly in overweight or obese patients, plays a crucial role in reducing symptoms and slowing OA progression. Even modest weight loss (5–10%) results in meaningful improvements [53,54,55,56,57,58]. Patient education, including guidance on joint protection, footwear, and flare management, is essential; however, more research is needed to establish optimal strategies [54,57,58]. Additional options such as cognitive behavioral therapy, bracing, acupuncture, and thermal interventions may be beneficial for selected patients [54]. These combined strategies address not only physical symptoms but also support long-term self-management and prevention.

#### 4.4.2. Pharmacological Management

Topical nonsteroidal anti-inflammatory drugs (NSAIDs) are recommended as the first pharmaceutical therapy, and oral administration is recommended only after that. In advanced stages, Intra-articular glucocorticoid injections (with ultrasound guidance) are strongly recommended for knee OA sufferers [59]. Additionally, acetaminophen, duloxetine, and tramadol are conditionally recommended for pain management in knee OA, with increased medical supervision, especially regarding opioids [60]. Moreover, despite previous indications, administering bisphosphonates, glucosamine, Chondroitin sulfate, hydroxychloroquine, and methotrexate is not recommended as a therapeutic management [54].

Curcumin seems to have good clinical efficacy and safety as a supplement in knee OA treatment, especially in combination with other therapies (NSAIDs) [61].

While intra-articular hyaluronic acid injection is not recommended for hip osteoarthritis, the therapy appears to be moderately effective in pain management in the early to moderate stages of knee OA [54,62,63]. Despite previous results, recent studies suggest that platelet-rich plasma treatment in knee osteoarthritis (OA) may provide pain relief, improve function, and decrease stiffness, particularly in mild to moderate knee OA (KL 1–3) [64].

Comorbidities such as hypertension, anxiety, and depression should be addressed according to established guidelines, recognizing that the effects of osteoarthritis-related impairment may be intensified by the presence of these conditions [59,65]. Additionally, in the realm of obesity management, particularly considering the increased weight-bearing on joints, metformin—which has been extensively investigated over recent decades for its diverse effects beyond glycemic control—has recently been proposed as a potentially effective treatment for symptomatic knee osteoarthritis in individuals who are overweight or obese [66].

Rather than treating all OA patients homogenously, our study supports a stratified, phenotype-based management approach. Each of the four clusters identified by K-means analysis presents distinct clinical profiles that may inform personalized care pathways, and propose different methods based on the clinical OA stage:

Cluster 1 (Mild OA): Early-stage patients who may benefit from preventive strategies—weight management, exercise, and education.

Cluster 2 (Moderate OA): Candidates for conservative pharmacologic therapy and physiotherapy.

Cluster 3 (Moderate-Severe OA): Likely to require multidisciplinary care and evaluation for potential surgical intervention.

Cluster 4 (Severe OA–Depressive–Metabolic phenotype): These complex patients may benefit most from integrative care, including rheumatologic, psychological, and metabolic interventions.

This stratified approach aligns with the current paradigm shift in OA management, from structure-based treatment to phenotype-driven therapy. Our results underscore the clinical utility of combining the WOMAC score, KL grade, and comorbidity profiles to inform individualized management, enhance outcomes, and ensure patient safety.

*Clinical implications*: This study highlights the importance of integrating patient-reported outcomes, radiographic assessments, and comorbidity profiling to inform personalized management in knee osteoarthritis. The proposed WOMAC cut-off values aligned with KL grades offer a practical framework for stratifying disease severity and tailoring interventions. Identifying distinct OA phenotypes—particularly those with psychological and metabolic comorbidities—supports a move toward multidimensional, individualized care plans rather than uniform treatment protocols. This approach may improve therapeutic efficacy, optimize resource allocation, and enhance patient safety in clinical practice.

*Strengths of the research*: This study supports the concept of WOMAC sub-phenotyping by identifying distinct osteoarthritis phenotypes based on symptom severity, functional impairment, and radiographic grading. These findings emphasize the gap between structural damage and patient symptoms, highlighting the need for a multidimensional OA classification system. This approach has clinical relevance, as it aligns different phenotypes with personalized treatment strategies, ranging from prevention to advanced interventions.

*Limitations of the research*: This study has several limitations. First, the statistical power for multiclass ROC analyses was limited at the extremes of the KL spectrum, particularly for KL = 4, where small group sizes resulted in wider confidence intervals and greater uncertainty. Second, the single-center outpatient sample introduces potential spectrum bias, which may limit the generalizability of the findings to broader or more diverse populations. Third, the cut-off values and clustering solution have not been externally validated. Although we performed internal optimism correction using the 0.632 bootstrap method and confirmed threshold stability across resamples, external test sets or multicenter validation are needed to verify reproducibility.

Additional considerations include the fact that KL grading may not fully capture functional burden, as structural changes do not always correspond to patient-reported outcomes. Advanced imaging and integration of biological markers could provide more refined phenotyping. Moreover, while K-means clustering offered an interpretable solution, alternative machine learning approaches might yield different subgroup structures. Finally, as the cross-sectional design precludes causal inference, prospective interventional trials will be necessary to validate whether the identified thresholds and clusters can meaningfully guide personalized treatment strategies.

Despite these limitations, the study demonstrates the feasibility of WOMAC-based phenotyping and threshold derivation, offering a foundation for future research aimed at refining OA classification and supporting more individualized clinical decision-making.

*Future Research Directions*: Given the cross-sectional design and single-center nature of this study, future research needs to focus on multi-center longitudinal studies to validate the proposed WOMAC cut-off values and phenotypes over time. Prospective cohort studies could monitor changes in symptoms and radiographic features, providing a clearer understanding of disease progression and the temporal relationship between structural changes and symptoms. Furthermore, randomized controlled trials (RCTs) that target interventions based on phenotype could assess whether stratified, individualized management yields better outcomes compared to conventional methods. Incorporating advanced imaging techniques and biomarkers, such as MRI and inflammatory cytokines, may further enhance phenotyping efforts. Lastly, by integrating patient-reported outcomes, comorbidities, and objective functional assessments into electronic health records, we could facilitate large-scale, real-world validation of these findings and support the development of predictive algorithms for clinical applications.

## 5. Conclusions

This exploratory study suggests that thresholds based on the WOMAC scale, along with radiographic and psychological variables, can categorize patients with knee OA into clinically distinct subgroups. While this approach showed internal consistency through bootstrap validation, its findings should be interpreted with caution due to the cross-sectional design, moderate sample size, and single-center recruitment. Further multicenter and longitudinal studies are needed to confirm the stability of these thresholds and clustering methods. If validated, such stratification tools could be integrated into routine clinical practice to support individualized treatment planning, guide patient selection for rehabilitation or pharmacological interventions, and enhance shared decision-making between clinicians and patients.

## Figures and Tables

**Table 1:** Participants’ characteristics.

Characteristics	Mean ± SD/n (%)
Age (years)	52.84 ± 15.22
BMI (kg/m^2^)	25.91 ± 5.61
Lequesne Index	6.30 ± 2.58
WOMAC Pain	12.74 ± 5.22
WOMAC Stiffness	5.09 ± 2.18
WOMAC Function	44.85 ± 15.18
WOMAC Total	62.75 ± 22.10
VAS Pain (0–10)	6.03 ± 2.14
HADS (total score)	7.74 ± 4.34
KL	2.35 ± 0.87
Joint space narrowing, grade (0–3)	1.61 ± 1
Joint space narrowing present, n (%)	84 (84.8%)
Osteophytes, grade (0–3)	1.67 ± 0.7
Osteophytes present, n (%)	94 (94.9%)
Hypertension (yes)	40 (40.4%)
Diabetes (yes)	20 (20.2%)

**Table 2:** WOMAC cut-off scores classification.

Category	WOMAC Score Interval	KL Grade Association	Specific Cut-Off (Youden J)	AUC (95% CI)	Clinical Interpretation
No symptoms	≤24	KL ≥ 1 vs. KL = 0	24	0.976 (0.938–1.000)	Minimal or no symptoms, no radiographic changes
Mild	25–41	KL ≥ 2 vs. KL ≤ 1	41	1.000 (1.000–1.000)	Mild symptoms, early structural joint changes
Moderate	42–69	KL ≥ 3 vs. KL ≤ 2	69	0.943 (0.892–0.980)	Clear pain and stiffness, moderate functional limitations
Severe	70–86	KL = 4 vs. KL ≤ 3	87	0.944 (0.832–1.000)	Severe pain and significant mobility loss
Extreme	≥87	KL 4	87	0.944 (0.832–1.000)	End-stage OA, high likelihood of disability.

Notes: Cut-offs obtained by Youden’s J from ROC analyses (DeLong AUCs). 95% CIs for sensitivity/specificity were computed with Wilson intervals. Full ROC metrics are provided in Supplementary Table S1.

**Table 3:** Descriptive analysis of each cluster’s characteristics, Mean, and SD.

Variable	1 Mild OA (n = 22)	2 Moderate OA (n = 29)	3 Moderate-Severe OA (n = 19)	4 Severe OA (n = 29)
KL Grade	0–2	2–3	3	3–4
WOMAC Total	29.15 ± 9.18	56.81 ± 8.73	71.83 ± 7.22	88.64 ± 4.55
WOMAC Pain	5.45 ± 2.35	10.74 ± 2.08	14.74 ± 2.07	19.20 ± 1.00
WOMAC Stiffness	2.25 ± 1.25	4.29 ± 1.24	5.91 ± 1.04	7.60 ± 0.71
WOMAC Functional	21.45 ± 6.59	41.58 ± 6.85	51.13 ± 5.22	61.84 ± 3.79
Joint Space Narrowing	0.25 ± 0.44	1.23 ± 0.50	2.48 ± 0.51	2.40 ± 0.50
Osteophytes	0.85 ± 0.59	1.32 ± 0.48	2.22 ± 0.42	2.24 ± 0.44
Lequesne Index	2.40 ± 0.68	5.48 ± 0.85	7.65 ± 0.98	9.20 ± 0.96
BMI	21.24 ± 2.41	23.95 ± 3.75	30.38 ± 5.30	27.97 ± 5.82
Age (Years)	31.30 ± 8.30	50.58 ± 8.74	59.91 ± 8.58	66.36 ± 9.83
Pain (VAS)	3.00 ± 0.86	5.16 ± 0.73	7.00 ± 0.74	8.64 ± 0.57
HADS	1.75 ± 1.77	6.77 ± 1.63	10.26 ± 2.96	11.40 ± 3.46
Hypertension %	0.00%	19.35%	69.57%	72.00%

## Data Availability

The data are available upon request from the corresponding author.

## Supplementary Materials

The following supporting information can be downloaded at https://www.mdpi.com/article/10.3390/life15101542/s1, Figure S1. Standardized cluster profiles of clinical and radiographic variables across four knee osteoarthritis subgroups derived from K-means clustering; Table S1. Receiver operating characteristic (ROC) analysis of WOMAC total scores against Kelgren-Lawrence (KL) grades: AUCs, sensitivity, specificity and 95% Cls; Table S2. Cluster stability and internal validity metrics: silhouette coefficients and Jaccard similarity indices for four K-means groups; Table S3. One way ANOVA and post-hoc comparisons for between-cluster differences in WOMAC subscores, Lequesne Index, VAS, HADS, BMI, age and radiographic features; Table S4. Chi-square associations and standardized residuals for categorical variables (e.g., hypertension distribution) across severity clusters; Table S5. Bootstrap validation of ROC analyses: apparent vs. optimism corrected AUCs and stability of Youden thresholds (1,000 resamples); Table S6. Post-hoc pairwaise comparisons for all clinical variables across K-means severity clusters (Hedges.s effect sizes included).
