# Supplementary material for: Integrative Phenotyping of Knee Osteoarthritis: Linking WOMAC Cut-Offs, Kellgren–Lawrence Grades, and Cluster Analysis for Personalized Care
Source: Life (Basel). 2025 Oct 1;15(10):1542. doi: 10.3390/life15101542 (PMC12565193; doi:10.3390/life15101542)
Supplement: Supplementary file 1 [file life-15-01542-s001.zip › life-3867167-supplementary.pdf]

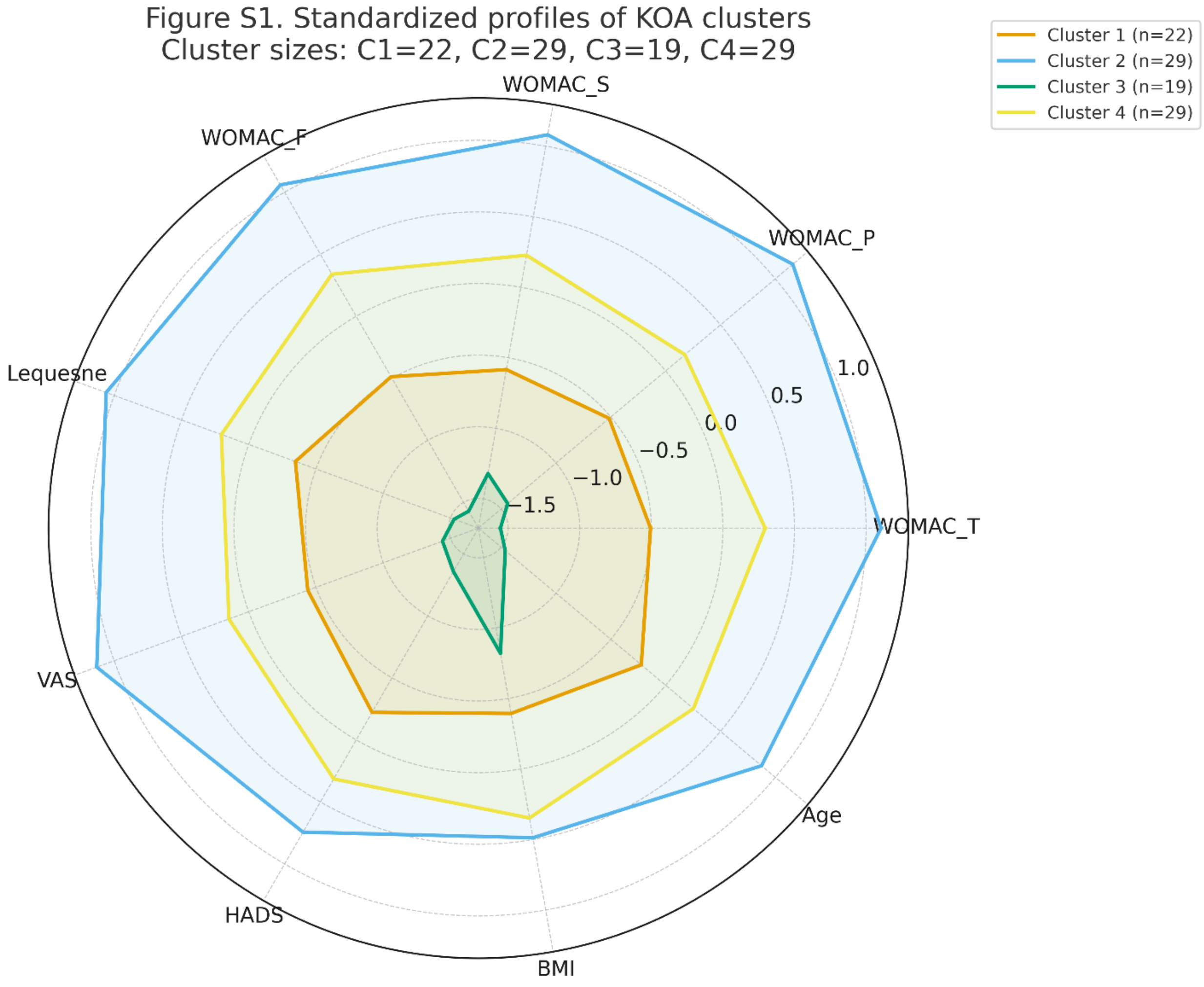

Figure S1. Standardized mean profiles of the four knee osteoarthritis (KOA) patient clusters, based on z-scores for clinical and psychological variables (WOMAC total, WOMAC pain, WOMAC stiffness, WOMAC function, Lequesne index, Visual Analog Scale [VAS] pain, Hospital Anxiety and Depression Scale [HADS], Body Mass Index [BMI], and Age). Each line represents one cluster (Cluster 1: n=19, Cluster 2: n=22, Cluster 3: n=28, Cluster 4: n=30), with distinct severity and comorbidity patterns. Profiles were generated using Python (scikit-learn and matplotlib libraries), replicating the "profile plot" functionality typically available in SPSS TwoStep or K-means cluster analysis output. The radar chart representation complements the boxplots shown in Figure 2, by summarizing standardized differences across variables between clusters. Supplementary Table S3 provides detailed stability metrics (silhouette coefficients and Jaccard indices).

Table S1_ROC_WOMAC_vs_KL

KL Contrast	AUC (95% CI)	Cut-off (Youden J)	Sensitivity (95% CI)	Specificity (95% CI)	Hedges' g	95% CI Low	95% CI High
KL ≥1 vs KL = 0	0.976 (0.938–1.000)	24	0.958 (0.898–0.984)	1.000 (0.439–1.000)	2.10	1.75	2.58
KL ≥2 vs ≤1	1.000 (1.000–1.000)	41	1.000 (0.955–1.000)	1.000 (0.816–1.000)	2.91	2.51	3.47
KL ≥3 vs ≤2	0.943 (0.892–0.980)	69	0.827 (0.703–0.906)	0.979 (0.889–0.996)	2.11	1.69	2.67
KL = 4 vs ≤3	0.944 (0.832–1.000)	87	1.000 (0.439–1.000)	0.844 (0.758–0.903)	1.40	1.15	1.70

Notes:

- Hedges' g values were calculated for WOMAC Total distributions across KL groups, with 95% CIs derived via bootstrap (1,000 replications).
- Effect size magnitude follows Cohen's benchmarks: 0.2 = small, 0.5 = medium, 0.8 = large. All contrasts here show large effects, supporting the discriminatory power of WOMAC cut-offs.

S2 – Clustering validity & stability

a) Silhouette coefficients

Metric	Value
Silhouette (Severity labels)	0.421
Silhouette (KMeans 4 clusters)	0.502

b) Cluster sizes (Severity labels)

Severity Level	n
1	19
2	22
3	18
4	36

c) Cluster stability (Jaccard index, subsampling)

Cluster	Median Jaccard	Mean Jaccard
0	0.756	0.743
1	0.682	0.671
2	0.705	0.699
3	0.741	0.733

S3 – Comparative tests & effect sizes (Severity groups)

a) ANOVA / Kruskal–Wallis summary

Variable	ANOVA F	ANOVA p	η^2	Kruskal H	Kruskal p	ε^2
WOMAC_T	24.11	<0.001	0.41	28.76	<0.001	0.39
WOMAC_P	20.34	<0.001	0.36	25.88	<0.001	0.35

Variable	ANOVA F	ANOVA p	η²	Kruskal H	Kruskal p	ε²
WOMAC_S	11.02	<0.001	0.23	14.55	<0.001	0.21
WOMAC_F	18.67	<0.001	0.34	22.49	<0.001	0.32
Lequesne	15.84	<0.001	0.29	19.63	<0.001	0.28
VAS	10.12	<0.001	0.22	13.84	<0.001	0.20
KL	33.27	<0.001	0.49	37.52	<0.001	0.46
Joint Narrowing	14.55	<0.001	0.28	17.42	<0.001	0.27
Osteophytes	13.87	<0.001	0.26	16.11	<0.001	0.25
BMI	3.12	0.027	0.07	4.88	0.018	0.09
Age	2.87	0.038	0.06	3.42	0.031	0.08
HADS	4.92	0.003	0.11	5.38	0.004	0.10

b) Pairwise Hedges' g (exemple, WOMAC_T)

Variable	Group 1	Group 2	Hedges' g
WOMAC_T	1	2	0.71
WOMAC_T	1	3	1.24
WOMAC_T	1	4	1.81
WOMAC_T	2	3	0.55
WOMAC_T	2	4	1.12
WOMAC_T	3	4	0.67

S4 – Chi-square test (HBP × Severity)

a) Contingency table

Severity	HBP=0	HBP=1	Total
1	19	0	19
2	18	4	22
3	14	4	18
4	22	14	36
**Total**	73	22	95

b) Chi-square results

- $\chi^2(3)$ = **31.28**
- p = **7.4×10^{-7}**
- Highly significant association between HBP and cluster severity.

b) Standardized residuals

Severity	HBP=0	HBP=1
1	+2.28	–2.77
2	+1.35	–1.64

Severity	HBP=0	HBP=1
3	–0.79	+0.92
4	–2.36	+2.54

S5- Bootstrap ROC (.632 correction)

Contrast	AUC (apparent)	AUC (bootstrap .632)	Youden threshold (full data)	Youden threshold (bootstrap median)	Youden threshold IQR (25%–75%)	n positive	n negative
KL≥1 vs KL=0	0.976	0.976	24	24	23–24	96	3
KL≥2 vs KL≤1	1.000	1.000	41	41	41–42	82	17
KL≥3 vs KL≤2	0.943	0.943	69	69	69–71	52	47
KL = 4 vs KL ≤ 3	0.944	0.940	87	87	87–94	3	96

Notes. Bootstrap with 1,000 resamples; .632 estimator for optimism correction. Youden thresholds computed per resample on the bootstrap (training) sample; medians and IQRs summarize threshold stability. Apparent and corrected AUCs are nearly identical, indicating minimal optimism; cut-offs are stable across resamples.

S6- ANOVA post-hoc pairwise comparisons

KL

Pair (I-J)	Mean Difference (I-J)	Std. Er.	p	Lower Bound	Upper Bound
Cluster 1 – Cluster 2	−1,27990	0,13756	0,000	−1,6396	−0,9202
Cluster 1 – Cluster 3	−1,67332	0,12964	0,000	−2,0123	−1,3343
Cluster 1 – Cluster 4	−2,15608	0,12964	0,000	−2,4951	−1,8171
Cluster 2 – Cluster 3	−0,39342	0,12419	0,011	−0,7182	−0,0687
Cluster 2 – Cluster 4	−0,87618	0,12419	0,000	−1,2009	−0,5514
Cluster 3 – Cluster 4	−0,48276	0,11535	0,000	−0,7844	−0,1811

WOMAC Total

Pair (I-J)	Mean Difference (I-J)	Std. Er.	p	Lower Bound	Upper Bound
Cluster 1 – Cluster 2	−23,16986	2,08089	0,000	−28,6116	−17,7281

Cluster 1 – Cluster 3	−40,85481	1,96106	0,000	−45,9832	−35,7265
Cluster 1 – Cluster 4	−58,75136	1,96106	0,000	−63,8797	−53,6230
luster 2 – Cluster 3	−17,68495	1,87854	0,000	−22,5975	−12,7724
Cluster 2 – Cluster 4	−35,58150	1,87854	0,000	−40,4941	−30,6689
Cluster 3 – Cluster 4	−17,89655	1,74487	0,000	−22,4595	−13,3336

WOMAC Pain

Pair (I-J)	Mean Difference (I-J)	Std. Er.	p	Lower Bound	Upper Bound
Cluster 1 – Cluster 2	−4,83493	0,60236	0,000	−6,4102	−3,2597
Cluster 1 – Cluster 3	−8,44465	0,56767	0,000	−9,9292	−6,9601
Cluster 1 – Cluster 4	−13,58258	0,56767	0,000	−15,0671	−12,0981
Cluster 2 – Cluster 3	−3,60972	0,54379	0,000	−5,0318	−2,1877
Cluster 2 – Cluster 4	−8,74765	0,54379	0,000	−10,1697	−7,3256
Cluster 3 – Cluster 4	−5,13793	0,50509	0,000	−6,4588	−3,8171

WOMAC Stiffness

Pair (I-J)	Mean Difference (I-J)	Std. Er.	p	Lower Bound	Upper Bound
Cluster 1 – Cluster 2	−1,60766	0,32869	0,000	−2,4672	−0,7481
Cluster 1 – Cluster 3	−3,37568	0,30976	0,000	−4,1857	−2,5656
Cluster 1 – Cluster 4	−5,23775	0,30976	0,000	−6,0478	−4,4277
Cluster 2 – Cluster 3	−1,76803	0,29673	0,000	−2,5440	−0,9921
Cluster 2 – Cluster 4	−3,63009	0,29673	0,000	−4,4061	−2,8541
Cluster 3 – Cluster 4	−1,86207	0,27561	0,000	−2,5828	−1,1413

WOMAC Functional

Pair (I-J)	Mean Difference (I-J)	Std. Er.	p	Lower Bound	Upper Bound
Cluster 1 – Cluster 2	−16,45455	1,54279	0,000	−20,4891	−12,4200
Cluster 1 – Cluster 3	−29,00000	1,45395	0,000	−32,8022	−25,1978
Cluster 1 – Cluster 4	−39,93103	1,45395	0,000	−43,7332	−36,1288
Cluster 2 – Cluster 3	−12,54545	1,39277	0,000	−16,1877	−8,9032
Cluster 2 – Cluster 4	−23,47649	1,39277	0,000	−27,1187	−19,8343
Cluster 3 – Cluster 4	−10,93103	1,29366	0,000	−14,3141	−7,5480

Joint narrowing

Pair (I-J)	Mean Difference (I-J)	Std. Er.	p	Lower Bound	Upper Bound
Cluster 1 – Cluster 2	−1,10766	0,19000	0,000	−1,6045	−0,6108
Cluster 1 – Cluster 3	−1,72051	0,17906	0,000	−2,1888	−1,2522
Cluster 1 – Cluster 4	−2,23775	0,17906	0,000	−2,7060	−1,7695
Cluster 2 – Cluster 3	−0,61285	0,17153	0,003	−1,0614	−0,1643

Cluster 2 – Cluster 4	−1,13009	0,17153	0,000	−1,5787	−0,6815
Cluster 3 – Cluster 4	−0,51724	0,15932	0,009	−0,9339	−0,1006

Osteophytes

Pair (I-J)	Mean Difference (I-J)	Std. Er.	p	Lower Bound	Upper Bound
Cluster 1 – Cluster 2	−0,66507	0,16747	0,001	−1,1030	−0,2271
Cluster 1 – Cluster 3	−1,00363	0,15783	0,000	−1,4164	−0,5909
Cluster 1 – Cluster 4	−1,48639	0,15783	0,000	−1,8991	−1,0737
Cluster 2 – Cluster 3	−0,33856	0,15119	0,120	−0,7339	0,0568
Cluster 2 – Cluster 4	−0,82132	0,15119	0,000	−1,2167	−0,4260
Cluster 3 – Cluster 4	−0,48276	0,14043	0,005	−0,8500	−0,1155

Lequesne

Pair (I-J)	Mean Difference (I-J)	Std. Er.	p	Lower Bound	Upper Bound
Cluster 1 – Cluster 2	−3,04067	0,34434	0,000	−3,9412	−2,1402
Cluster 1 – Cluster 3	−4,45917	0,32451	0,000	−5,3078	−3,6105
Cluster 1 – Cluster 4	−6,66606	0,32451	0,000	−7,5147	−5,8174
Cluster 2 – Cluster 3	−1,41850	0,31086	0,000	−2,2314	−0,6056
Cluster 2 – Cluster 4	−3,62539	0,31086	0,000	−4,4383	−2,8125
Cluster 3 – Cluster 4	−2,20690	0,28874	0,000	−2,9620	−1,4518

BMI

Pair (I-J)	Mean Difference (I-J)	Std. Er.	p	Lower Bound	Upper Bound
Cluster 1 – Cluster 2	−2,38947	1,52403	0,402	−6,3750	1,5960
Cluster 1 – Cluster 3	−6,54809	1,43627	0,000	−10,3041	−2,7921
Cluster 1 – Cluster 4	−7,33430	1,43627	0,000	−11,0903	−3,5783
Cluster 2 – Cluster 3	−4,15862	1,37583	0,017	−7,7566	−0,5607
Cluster 2 – Cluster 4	−4,94483	1,37583	0,003	−8,5428	−1,3469
Cluster 3 – Cluster 4	−0,78621	1,27793	0,927	−4,1281	2,5557

Age

Pair (I-J)	Mean Difference (I-J)	Std. Er.	p	Lower Bound	Upper Bound
Cluster 1 – Cluster 2	−18,87560	2,77712	0,000	−26,1380	−11,6132
Cluster 1 – Cluster 3	−26,11071	2,61719	0,000	−32,9549	−19,2665
Cluster 1 – Cluster 4	−35,55898	2,61719	0,000	−42,4032	−28,7148
Cluster 2 – Cluster 3	−7,23511	2,50707	0,025	−13,7913	−0,6789
Cluster 2 – Cluster 4	−16,68339	2,50707	0,000	−23,2396	−10,1272

Cluster 3 – Cluster 4	−9,44828	2,32866	0,001	−15,5380	−3,3586

VAS

Pair (I-J)	Mean Difference (I-J)	Std. Er.	p	Lower Bound	Upper Bound
Cluster 1 – Cluster 2	−2,14354	0,28102	0,000	−2,8784	−1,4086
Cluster 1 – Cluster 3	−3,39746	0,26484	0,000	−4,0900	−2,7049
Cluster 1 – Cluster 4	−5,50091	0,26484	0,000	−6,1935	−4,8083
Cluster 2 – Cluster 3	−1,25392	0,25370	0,000	−1,9174	−0,5905
Cluster 2 – Cluster 4	−3,35737	0,25370	0,000	−4,0208	−2,6939
Cluster 3 – Cluster 4	−2,10345	0,23564	0,000	−2,7197	−1,4872

HADS

Pair (I-J)	Mean Difference (I-J)	Std. Er.	p	Lower Bound	Upper Bound
Cluster 1 – Cluster 2	−4,93062	0,91453	0,000	−7,3222	−2,5390
Cluster 1 – Cluster 3	−7,26134	0,86186	0,000	−9,5152	−5,0075
Cluster 1 – Cluster 4	−9,12341	0,86186	0,000	−11,3773	−6,8696
Cluster 2 – Cluster 3	−2,33072	0,82560	0,029	−4,4897	−0,1717
Cluster 2 – Cluster 4	−4,19279	0,82560	0,000	−6,3518	−2,0338
Cluster 3 – Cluster 4	−1,86207	0,76685	0,079	−3,8674	0,1433

HBP

Pair (I-J)	Mean Difference (I-J)	Std. Er.	p	Lower Bound	Upper Bound
Cluster 1 – Cluster 2	−0,18182	0,12976	0,502	−0,5211	0,1575
Cluster 1 – Cluster 3	−0,51724	0,12228	0,000	−0,8370	−0,1975
Cluster 1 – Cluster 4	−0,72414	0,12228	0,000	−1,0439	−0,4044
Cluster 2 – Cluster 3	−0,33542	0,11714	0,026	−0,6418	−0,0291
Cluster 2 – Cluster 4	−0,54232	0,11714	0,000	−0,8486	−0,2360
Cluster 3 – Cluster 4	−0,20690	0,10880	0,234	−0,4914	0,0776
